# The Effect of Plasma Treatment on the Mechanical and Biological Properties of Polyurethane Artificial Blood Vessel

**DOI:** 10.3390/ma16227231

**Published:** 2023-11-19

**Authors:** Cheng Ding, Jing Ma, Yingxue Teng, Shanshan Chen

**Affiliations:** 1 School of Materials and Metallurgy, University of Science and Technology Liaoning, Anshan 114051, China; dc2019093001@163.com; 2 Shi-Changxu Innovation Center for Advanced Materials, Institute of Metal Research, Chinese Academy of Sciences, Shenyang 110016, China; jma@imr.ac.cn

**Keywords:** polyurethane, artificial blood vessels, electrospinning, plasma treatment, biocompatibility, mechanical properties

## Abstract

In recent years, the incidence of cardiovascular disease has increased annually, and the demand for artificial blood vessels has been increasing. Due to the formation of thrombosis and stenosis after implantation, the application of many materials in the human body has been inhibited. Therefore, the choice of surface modification process is very important. In this paper, small-diameter polyurethane artificial blood vessels were prepared through electrospinning, and their surfaces were treated with plasma to improve their biological properties. The samples before and after plasma treatment were characterized by SEM, contact angle, XPS, and tensile testing; meanwhile, the cell compatibility and blood compatibility were evaluated. The results show that there are no significant changes to the fiber morphology or diameter distribution on the surface of the sample before and after plasma treatment. Plasma treatment can increase the proportion of oxygen-containing functional groups on the surface of the sample and improve its wettability, thereby increasing the infiltration ability of cells and promoting cell proliferation. Plasma treatment can reduce the risk of hemolysis, and does not cause platelet adhesion. Due to the etching effect of plasma, the mechanical properties of the samples decreased with the extension of plasma treatment time, which should be used as a basis to balance the mechanical property and biological property of artificial blood vessels. But on the whole, plasma treatment has positive significance for improving the comprehensive performance of samples.

## 1. Introduction

With the continuous growth of population and the aggravation of aging, the number of people suffering from cardiovascular diseases is increasing, and cardiovascular diseases have become one of the main threats to human health [1,2,3,4,5]. According to the 2023 World Health Statistics Report, in 2019, the number of deaths caused by cardiovascular diseases in the world was as high as 17.9 million, and the number of deaths is expected to continue to rise in coming years.

Atherosclerosis is the main cause of cardiovascular disease [6,7,8]. An effective treatment of atherosclerosis is to implant vascular grafts to replace diseased blood vessels [9]. Among them, autologous blood vessels are a better choice as vascular grafts, because of their good autologous histocompatibility [10]. However, due to the fact that vascular grafts need to meet a series of conditions, such as inner diameter, length, etc., there are few autologous blood vessels that meet the requirements; artificial blood vessels have attracted much attention in the medical field in recent years due to their good designability [11,12,13,14,15,16,17,18].

Polyurethane materials have good biocompatibility, stability, and mechanical properties [19,20,21]. Micro–nano fiber materials have high porosity and a unique three-dimensional network structure, which can provide advantages for cell growth. Therefore, polyurethane fiber materials are often used as matrix materials for artificial blood vessels [22,23,24]. However, the occurrence of thrombosis and internal stenosis of blood vessels after artificial blood vessel implantation inhibit their use in the human body [25,26,27,28,29,30]. The rapid endothelialization of the surfaces of artificial blood vessels will greatly improve the above shortcomings [31]. At present, endothelialization and anti-thrombosis are mainly accelerated by material surface modification and drug loading [31,32]. Zhang, Yuanguo et al. [13] prepared the polyurethane/gelatin core–shell structure coaxial fiber vascular membrane, using the coaxial electrospinning method to coat gelatin on the surface of polyurethane fiber and then utilizing chemical crosslinking. The experimental results show that, compared with ordinary polyurethane artificial blood vessels, coaxial artificial blood vessels have a good vascular remodeling performance. Binh Vu et al. [33] prepared polyurethane/polycaprolactone (PU/PCL) membranes with conjugated linoleic acid (CLA) by electrospinnning. The experimental results showed that the antithrombotic properties of the materials were improved after CLA grafting. The grafting of CLA can promote cell proliferation and accelerate endothelialization.

Plasma treatment has the advantages of high efficiency, a simple process, and little influence on the properties of materials, and is widely used in the field of material surface modification [34,35,36]. S. De et al. [37] explored the effect of plasma treatment with a He atmosphere on cell proliferation on the surface of polyurethane. The experimental results show that He plasma treatment can improve the hydrophilicity and roughness of polyurethane surfaces and promote cell proliferation. Keshel, Saeed Heidari et al. [38] used the plasma treatment of polyurethane films in an oxygen atmosphere to improve cell adhesion and proliferation on the surface of the material. However, the effect of plasma treatment on the overall biocompatibility and mechanical properties of polyurethane fiber materials has not been systematically explored.

In this paper, the effects of plasma treatment on the mechanical properties and biocompatibility of polyurethane fiber materials were systematically investigated using surface morphology and chemical composition analysis, mechanical property experiments, cell proliferation, and blood experiments. This study will support a novel method of surface functionalization for artificial blood vessels, and provide some theoretical guidance for the application of plasma treatment to the surfaces of artificial blood vessels. Its ultimate purpose is to settle the restenosis problem, especially for small-diameter artificial blood vessels.

## 2. Materials and Methods

### 2.1. Sample Preparation

Polyurethane (PU, medical polyether type, Bayer, Germany) was dissolved in a mixed solution of tetrahydrofuran (THF, Tianjin Fuyv, Tianjin, China) and N, N-dimethylformamide (DMF, Fuyu Chemical, Tianjin, China) at a mass fraction of 10%, with the volume ratio of THF and DMF being 4:3. After the PU was fully dissolved, the spinning solution was injected into an electrospinning device (YFSP-T, Yunfan Technology, Tianjin, China) in order to prepare a PU fiber material. The spinning parameters were as follows: the spinning voltage was 12 kV, the solution flow rate was 0.03 mL/min, the needle diameter was 1.35 mm, the working distance was 10 cm, the receiver was a roller receiver with a diameter of 100 mm, and the rotate speed was 270 r/min.

After the preparation of the PU fiber material, it was cut into 10 mm × 10 mm and 40 mm × 10 mm samples. In order to fully remove the solvent, the sample was placed at room temperature for 24 h, and then it was ultrasonically cleaned with anhydrous ethanol (Fuyu Chemical, Tianjin, China) 3 times, after which the sample was dried at room temperature for 24 h.

The samples were placed in the working chamber of the plasma generator (CIF, CPC-B-13.56) for plasma treatment; the plasma working atmosphere was high-purity oxygen (Dalian Special Gas), the power was 60 W, and the sample treatment times were 30 s, 60 s, 90 s, and 120 s, respectively.

In this experiment, 5 groups of samples were divided into a group of samples that did not undergo plasma treatment (noted as 0 s), and 4 groups that underwent different durations of plasma treatment (noted as 30 s, 60 s, 90 s, and 120 s).

### 2.2. Material Characterization

The morphologies of the fibers before and after plasma treatment were observed through scanning electron microscopy (SEM, Inspect F50, FEI, Hillsboro, OR, USA), and the fiber diameter was measured using ImageJ (V 1.8.0, National Institutes of Health) software.

The wettability of the sample was evaluated with a drop shape analyzer (DSA100, Kruss, Germany); due to the porous surface of the sample, the water droplets continue to penetrate as the contact time between the water droplets and the surface of the sample increases. For this reason, we used a high-speed camera to observe the infiltration process of water droplets at a rate of 60 frames per second to compare the wettability of different samples.

The types and contents of oxygen-containing functional groups on the surface of the samples before and after plasma treatment were analyzed through X-ray photoelectron spectroscopy (XPS, Escalab-250, Thermo Fisher Scientific, Waltham, MA, USA). Avantage (V 5.52, ThermoFisher Scientific) software was used to fit the XPS data.

The mechanical properties were tested with a tensile testing machine (Z050, Zwick, Ulm, Germany). The length of the tensile area of the sample was 20 mm, the width was 10 mm, and the tensile speed was 50 mm/min. The stress–strain curve of the sample was calculated from the original force–time curve, and the area with 0–100% deformation on the stress–strain curve was taken to calculate the Young’s modulus.

### 2.3. Cell Compatibility (In Vitro)

For evaluating the vascular response, human umbilical vein endothelial cells (HUVECs) were used for co-culture. According to GB/T 16886.5–2017 [39], the effects of different samples on cell proliferation rate were evaluated through the co-culture method. Some samples were used to observe the cell morphology on the surface of the samples. The specific operations are as follows:

The samples were placed on a sterile operating table for irradiation and sterilization with an ultraviolet lamp for 0.5 h. After sterilization, the samples were placed in 24-well plates, 600 μL of medium was added to each well plate, and then 400 μL of 50,000 cell/mL cell suspension was added. For the blank control group, medium and cell suspension were added into the well without samples; the other operation was the same as the sample group. The cell suspension in the well plate was mixed and allowed to stand for 5 min. Then, the well plate was placed in a constant temperature and humidity incubator at 37 °C and 5% carbon dioxide concentration for co-culture. After 24 h of co-culture, CCK-8 (cell counting kit-8) was added to the well plate with 10 μL per well, and the well plate was placed in the CO_2_ incubator again. After 4 h, the liquid in the 100 μL well plate was absorbed, and the absorbance (O.D.) was measured using a microplate reader with a light source wavelength of 450 nm. The Relative Growth Rate (RGR) of each group was calculated according to Formula (1). In the formula, O.D._t_ is the absorbance of the sample group, and O.D._CK_ is the absorbance of the control group.

(1) $$RGR=\frac{{O.D.}_{t}}{{O.D.}_{CK}}\times 100\%$$

The samples used to observe the surface cell morphology were co-cultured for 28 h, and the liquid in the orifice plate was sucked out; next, PBS (Phosphate Buffer Solution) was added to gently rinse and remove the cells floating on the surface of the sample. After PBS was removed, 2.5% glutaraldehyde solution was added, and the well plate was placed in a refrigerator at 4 °C for at least 2 h to fix the cells on the surface of the sample. Subsequently, 50%, 75%, 95%, and 100% ethanol solution was used to dehydrate step by step, and then the sample was taken out and dried at room temperature for 24 h. After spraying gold, the cell morphology on the surface of the sample was observed through scanning electron microscopy.

### 2.4. Blood Compatibility (In Vitro)

The blood compatibility test was divided into a hemolysis test and a platelet adhesion test in our study. The blood used in this experiment was donated by laboratory volunteers. The samples were rinsed with 75% alcohol in turn, rinsed with ionic water, and then rinsed twice with PBS, then drained for later use.

Fresh human blood was collected in an anticoagulant blood collection tube, and then the above anticoagulant blood was mixed with PBS solution at a volume ratio of 4:5.

Hemolysis experiment operation method: First, the sample was loaded into the test tube, and 0.8 mL PBS was added to the test tube as the experimental group sample.

Negative control group: 0.8 mL PBS was added into the tube. Positive control group: 0.8 mL deionized water was added to the test tube.

(2) $$Hemolysis\;rate=\frac{{O.D.}_{t}-{O.D.}_{nc}}{{O.D.}_{pc}-{O.D.}_{nc}}\times 100\%$$

Platelet adhesion assay: 2.5% glutaraldehyde solution was prepared with PBS. Anticoagulant blood was centrifuged at 1500 r/min for 20 min, and the supernatant was taken to prepare human PRP (Platelet-Rich Plasma). The test tube was added with 1 mL PRP to cover the sample, and then the test tube was placed in a constant temperature incubator at 37 °C for 60 min. After reaching the predetermined time, PRP in the test tube was aspirated and immediately supplied with PBS to gently rinse; the subsequent steps to observe the surface cell morphology are the same as the preparation steps for the sample, noted in Section 2.3.

### 2.5. Statistical Analysis

The data were shown as mean ± standard deviation (SD). Differences between groups were analyzed using one-way analysis of variance (ANOVA), and *p* < 0.05 was considered statistically significant.

## 3. Results and Discussion

### 3.1. Surface Morphology

**Figure 1:**
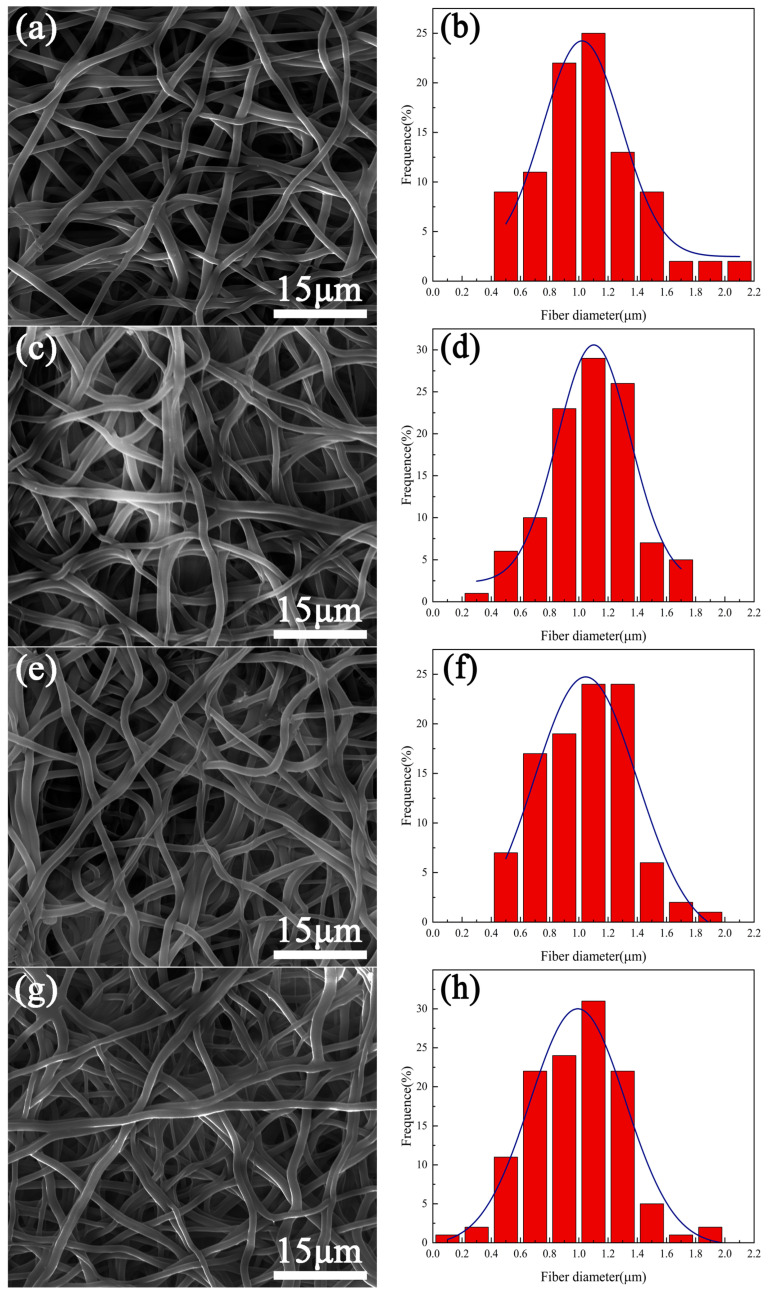

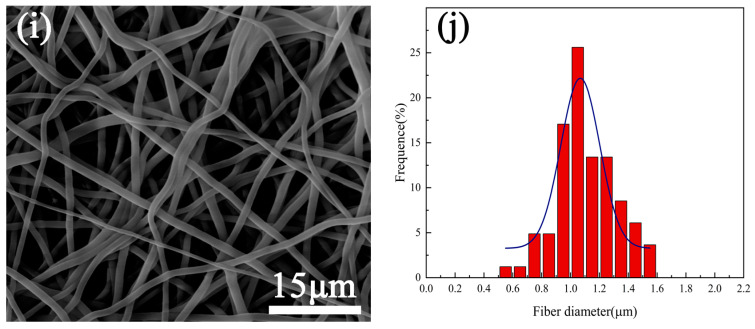
Fiber morphology and measured results of fiber diameter on the surface of the samples with plasma processed for different time periods—(**a**,**b**) 0 s, (**c**,**d**) 30 s, (**e**,**f**) 60 s, (**g**,**h**) 90 s, (**i**,**j**) 120 s.

Figure 1 shows the surface morphologies of the fiber samples without plasma treatment and those treated with plasma for 30 s, 60 s, 90 s, and 120 s. The histogram is the statistical result of the fiber diameter of each sample. It was found that each sample comprised numerous stacked fibers that formed a three-dimensional network structure. The fibers were randomly distributed and the porosity was high. From the statistical results, it can be seen that the fiber diameters of each group of samples are approximately concentrated between 0.4–1.6 μm; the fiber morphology of the samples did not change significantly with the extension of plasma treatment time. The peak of the fitting curve in the statistical diagram did not move significantly, which indicated that the plasma treatment with a duration of 120 s had little effect on the fiber diameter.

### 3.2. Wettability of Sample Surface

**Figure 2:**
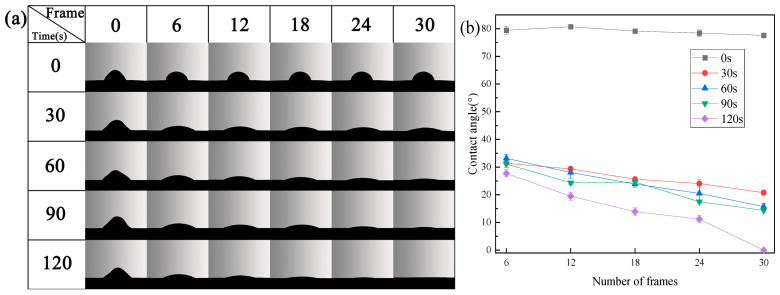
Water contact angle of PU samples after different periods of plasma treatment; (**a**) instantaneous photos, (**b**) measured results of contact angle.

Figure 2a shows the instantaneous contact of water droplets with each sample under different plasma treatment times. Figure 2b shows the results of the instantaneous contact angle between each sample surface and the corresponding water droplet.

Due to the porous surfaces of the samples, water droplets cannot exist stably on their surfaces. Therefore, we characterize the wetting ability of each sample by calculating the instantaneous contact angle, the value of the instantaneous contact angle at the same time, or the rate of change between the instantaneous contact angle and the contact time.

It can be seen from Figure 2 that, in each frame, the contact angle of the untreated sample is much greater than that of the samples treated with plasma; and the reduction rate of the contact angle of the water droplets on the surface of the sample is much smaller than that of the samples treated with plasma, which indicates that the wettability of the untreated sample is very poor and much smaller than that of the samples that underwent plasma treatment. With the extension of plasma treatment time, the contact angle of water droplets on the sample surface decreases with time, which indicates that the wettability of the sample surface increases with the extension of plasma treatment time.

Plasma treatment can significantly enhance the wettability of the sample, which is continuously improved with the extension of the plasma treatment time. In order to explain the reason why plasma treatment improves the wettability of the material surface, this paper presents a new method to improve material surface wettability.

The surface chemical composition of the samples was analyzed.

### 3.3. Surface Chemical Composition

**Figure 3:**
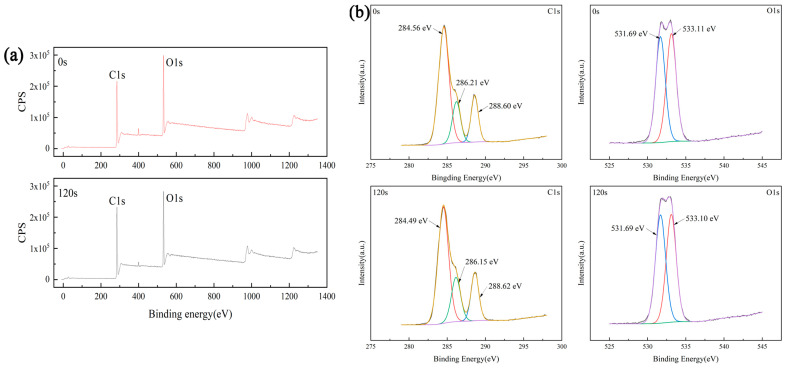
(**a**) XPS spectrum of untreated samples (0 s) and treated with plasma for 120 s; (**a**) full spectrum (CPS, Count Per Second), (**b**) peak fitting results.

In order to explain why the above plasma treatment can improve the wettability of the sample, XPS was used to analyze the surface compositions of both the untreated sample (0 s) and the sample treated with plasma for 120 s. Figure 3a is the full XPS spectrum of each test sample. The proportions of C and O elements on the surfaces of the samples are listed in Table 1. It can be seen that the proportion of oxygen on the surface of the sample treated with plasma is 29.02%, which is higher than that of the untreated sample (27.60%).

Figure 3b shows the fine spectra of C1s and O1s on the sample surface and the fitting results. Among them, the spectra of C1s can be fitted into three peaks, and their binding energies are 284, 286, and 288 eV, one for each of C–C, C–O, and C=O bonds, respectively. The spectra of O1s can be fitted into two peaks, and their binding energies are 531 and 533 eV for C–O bond and C=O bond, respectively. Table 2 shows the integral area of oxygen-containing functional groups. From Table 2, it can be seen that after plasma treatment, the integral area of C–O decreased from 126,101.08 to 128,696.42, while the integral area of C=O increased from 120,234.91 to 131,748.05.

The above results show that plasma treatment can increase the proportion of oxygen elements on the surface of PU. This is because oxygen is converted into free electrons and free radical particles with high activity during glow discharge based on the plasma formation mechanism. These free electrons and free radical particles impact the surface of the sample, destroy the original chemical bonds to produce new free electrons and free radical particles, and then form new chemical bonds with the destroyed chemical bonds, thus generating oxygen-containing functional groups on the surface of the sample. The increase in the generation of oxygen-containing functional groups improves the wettability of the sample, which is consistent with previous research results [40,41].

### 3.4. Mechanical Properties

**Figure 4:**
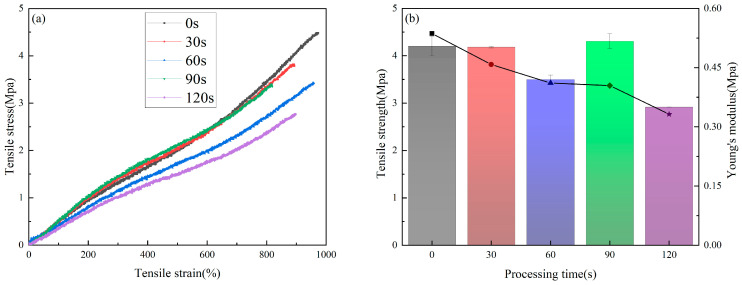
Tensile test results, (**a**) stress–strain curve, (**b**) Young’s modulus and ultimate tensile strength (the bar chart represents the Young’s modulus, and the scatter chart represents the tensile strength).

Figure 4a is the tensile curve of the samples treated by plasma for different time periods. It can be seen that the ultimate tensile strength of the untreated sample is 4.5 MPa, which is significantly higher than that measured in the samples subjected to plasma treatment. With the extension of the plasma treatment time, the tensile strength of the sample decreases continuously. When the treatment time is 120 s, the tensile strength of the sample decreases by about 40%, to 2.7 MPa.

In this study, the slope of the stress–strain curve in the range of 0–100% is used to calculate the Young‘s modulus, as shown in Figure 4b. The histogram corresponds to the changes in the Young‘s modulus of the samples, and the line chart corresponds to the changes in the tensile strength of the samples. The Young‘s modulus of the untreated sample was calculated to be 0.50 MPa, and the Young‘s modulus of the sample treated by plasma for 120 s decreased to 0.35 MPa.

In summary, plasma treatment will reduce the tensile strength and Young’s modulus of the sample. Plasma treatment has a greater impact on the tensile strength of the sample and a smaller impact on the Young’s modulus. This tendency may be caused by plasma etching, which could lead to micro cracks on the surface of the fiber, detailed in a previous paper [42]. With the extension of treatment time, the degree of cracking also increases. Due to the large specific surface area and porosity increase of the sample, the fiber inside the sample will also be etched by the plasma gas, which will eventually lead to a significant reduction in its mechanical properties.

C. M. García-Herrera et al. [43] found that the tensile strength of the ascending aorta in a healthy young person is about 2.18 Mpa, which is lower than the tensile strength of the sample treated with plasma for 120 s (about 2.7 Mpa). Simply considered from the point of view of mechanical properties, this indicated that the tensile strength of the sample treated with plasma for 120 s could make it a suitable substitute for the ascending aorta of a healthy young person. Combined with the test results of mechanical properties and the safety margin of material use, it is not recommended that the treatment time should be more than 120 s for polymer materials.

### 3.5. Biocompatibility Evaluation In Vitro

#### 3.5.1. Cell Experiment

**Figure 5:**
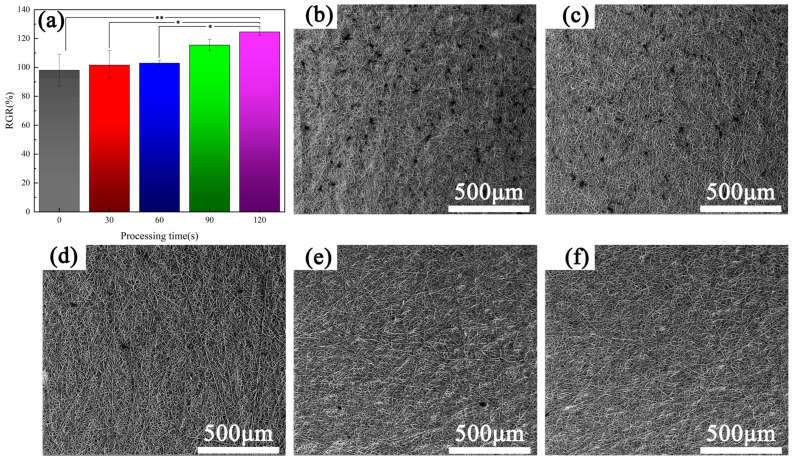
Results of cell proliferation assay (* *p* < 0.05, ** *p* < 0.01). (**a**) Relative Growth Rate (RGR); the surface morphology of each sample after co-culture for (**b**) 0 s, (**c**) 30 s, (**d**) 60 s, (**e**) 90 s, (**f**) 120 s.

Figure 5a shows the results of the cell proliferation experiment performed after one day of co-culture of endothelia cells and samples. It can be seen that the cell proliferation rate of the untreated sample was about 100%. After plasma treatment, the cell proliferation rate increased to a certain extent, and with the prolongation of plasma treatment time, the cell proliferation rate also increased. This is because the plasma treatment enhances the wettability of the sample, making it easier for cells to adhere and spread on the surface of the sample.

Figure 5b–f depict the scanning electron microscope images of each sample surface after one day of co-culture. The images show that the number of cells is significantly higher on the surface of the sample that did not undergo plasma treatment than on the surfaces of the plasma treated samples, and with the extension of the plasma treatment time, the number of cells on the surface of each sample decreases continuously. For the 90 s and 120 s plasma-treated samples, there are almost no cells on the surface of each sample; this is completely opposite to the results of the cell proliferation rate detected using the cck-8 method. This is because, with the extension of the plasma treatment time, the plasma atmosphere will spread deeper into the sample, the range of plasma treatment inside the sample will increase, and the wetting ability inside the sample will be improved. Compared with the surface of the sample, its internal surface area is larger; the excellent wetting ability and large surface area inside the sample will promote the migration and proliferation of endothelia cells. Therefore, with the prolongation of plasma treatment time, the proliferation ability of cells in the sample is increased, but the cells will be more likely to migrate and differentiate into the sample. As a result, when we extended the plasma treatment time, the number of cells on the surface of the samples decreased, which was an ‘abnormal’ phenomenon.

Careful observation of the surface of the samples after 90 s and 120 s of plasma treatment showed that there were a large number of protrusions on the surface, which may be caused by the proliferation of cells inside the samples. The morphology of cells is mainly observed through scanning electron microscopy and fluorescence microscopy, which are used to observe the adhesion of cells on the surface of the sample. For the sample with a network structure, the adhesion of cells in the mesh is difficult to observe. During the use of SEM in this study, no cells were observed migrating to the sample network, only regular convex structures were observed.

#### 3.5.2. Blood Test Results

In order to systematically study the effect of plasma treatment on the biocompatibility of polyurethane, the cell proliferation experiment of vascular endothelial cells, the hemolysis experiment, and the platelet adhesion experiment were carried out to evaluate the biocompatibility of the samples.

Hemolysis rate is an important index used to evaluate the blood compatibility of materials. According to ISO 10993-4 [44], the hemolysis rate of biomedical materials should be less than 5%, otherwise it indicates that the materials induce a hemolysis effect.

**Figure 6:**
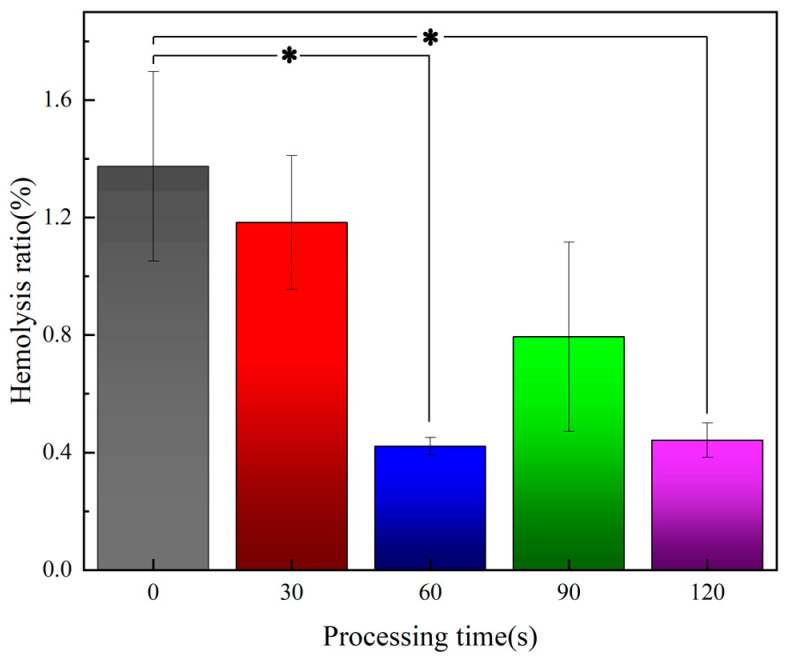
Hemolysis test results of samples (* *p* < 0.05).

Figure 6 shows the hemolysis test results of samples treated with plasma over different periods of time. It can be seen that the hemolysis rate of all samples was less than 5%, which indicates that all samples are not at risk of hemolysis; and the hemolysis rate of the samples reaches the lowest value after 60 s of plasma treatment, which indicates that the polyurethane samples have a certain effect on reducing the hemolysis rate after appropriate plasma treatment.

Platelet adhesion on the surface of the material is another important index needed to evaluate the blood compatibility of the material. The diameter of platelets is generally 7–8 μm, and platelets are disc-shaped; activated platelets produce protrusions that easily adhere to the surface of the material. Excessive adhesion of platelets on the surface of the material is the main cause of thrombosis.

**Figure 7:**
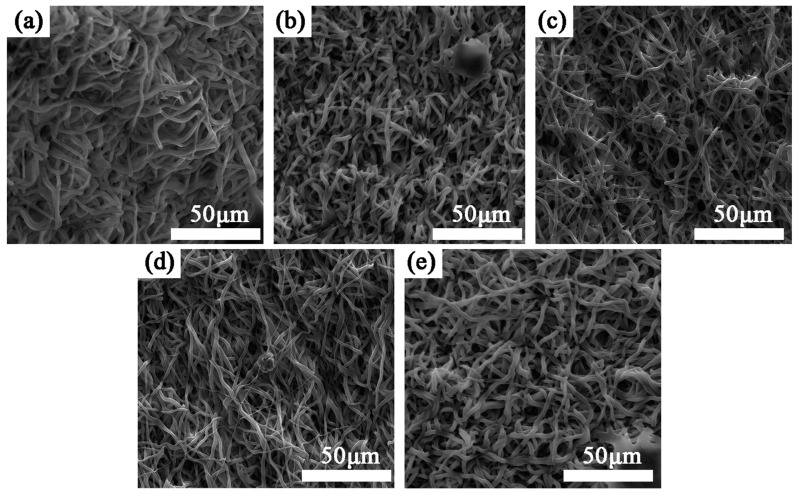
Results of platelet adhesion test. Samples were treated for (**a**) 0 s, (**b**) 30 s, (**c**) 60 s, (**d**) 90 s, (**e**) 120 s.

In this study, samples with different plasma treatment times were placed in PRP and incubated for 1 h and 3 h, respectively. Figure 7 shows the results of the platelet adhesion experiment (3 h). Scanning electron microscopy showed that there were no platelets on the surface of all samples, which indicated that the adhesion ability of platelets on the surface of samples was very weak both without plasma treatment and after plasma treatment. The samples under each treatment condition could resist platelet adhesion and prevent thrombus formation on their surfaces. Martina Modic et al. [45] found that plasma treatment can cause microcracks on the surfaces of polymer materials. Due to the small size of the crack, it will not have a significant impact on the adhesion behavior of platelets on the material surface. Due to their small size, plasma proteins—unlike platelets—are subject to microcracks providing active sites on their surfaces, covering the material surface with a layer of plasma protein film, which prevents platelets from adhering to the material surface. Based on the research results of this article and previous studies, it is demonstrated that materials without plasma treatment have excellent antiplatelet properties, and plasma treatment can further improve this performance.

## 4. Conclusions

In this paper, the effect of plasma treatment on the properties of polyurethane fiber samples was systematically studied for the first time. Under the premise of not introducing other components, plasma treatment can improve the cell proliferation rate and reduce the risk of hemolysis. It is expected to combine other surface modification methods in order to prepare artificial blood vessel materials with better performance. The findings of this study can be summarized as follows:

1. Under the plasma treatment conditions selected for this experiment, plasma treatment did not cause significant changes in the morphology of polyurethane fibers.
2. Plasma treatment can introduce oxygen-containing functional groups, which can significantly improve the wettability of the sample and increase the proliferation rate of endothelia cells on the sample.
3. Plasma treatment can reduce the risk of hemolysis of the material, improve the anti-platelet adhesion properties. Overall, plasma treatment can improve the blood compatibility of polyurethane fiber materials.
4. Due to the etching effect of plasma, the mechanical properties of the sample decreased to a certain extent; this tendency to decrease should be used as the basis to balance the biological properties and the mechanical properties during the design of PU artificial blood vessels.
5. In view of the influence of plasma treatment on mechanical properties and biocompatibility, polyurethane samples treated with plasma for 90 s have good mechanical properties and biocompatibility, and they also improve the wettability of the sample surface, which lays a foundation for the preparation of subsequent functional coatings.

## Figures and Tables

**Table 1:** The proportion of C and O elements on the surface of untreated samples (0 s) and plasma treatment for 120 s.

Processing Time (s)	Element	Integral Area	Proportion of Each Element (%)
0	C1s	882,842.61	72.40
	O1s	858,638.52	27.60
120	C1s	861,275.60	70.98
	O1s	898,559.74	29.02

**Table 2:** Integral area of oxygen-containing functional groups after peak separation fitting.

Processing Time (s)	C–O Integral Area	Proportion of C–O (%)	C=O Integral Area	Proportion of C=O (%)
0	126,101.08	51.21	120234.91	48.79
120	128,696.42	49.44	131748.05	50.56

## Data Availability

Data are contained within the article.
